# The Protective Effect of Omeprazole on Vancomycin Cytotoxicity in HK-2 Cells and Renal Injury in Rats

**DOI:** 10.1155/bmri/3520935

**Published:** 2025-07-31

**Authors:** Jiaxu Wu, Xikun Wu, Wenli Li, Yakun Zhang, Zhiqing Zhang

**Affiliations:** Department of Pharmacy, The Second Hospital of Hebei Medical University, Shijiazhuang, Hebei, China

**Keywords:** HK-2 cells, intracellular concentration, omeprazole, rats, vancomycin

## Abstract

**Objective:** Vancomycin is the first-line treatment for *MRSA* infection, and high plasma concentration can cause nephrotoxicity. The aim of the study was to determine the correlation between intracellular vancomycin concentration and HK-2 cytotoxicity and explore omeprazole's protective effect.

**Methods:** The activity of HK-2 cells was detected, HPLC method was established and verified, and the vancomycin concentrations in the intracellular and extracellular fluids of HK-2 cells were determined. Western blot was used to investigate the expressions of P-glycoprotein (P-gp) and organic cation transporter-2 (OCT-2) transporters. Blood urea nitrogen (BUN), blood creatinine (CRE), *N*-acetyl-*β*-d-glucosaminidase (NAG), and kidney injury molecule-1 (KIM-1) of rats in the vancomycin group and drug combination group were determined; the kidney tissue pathological examination and renal injury score were performed.

**Results:** The HPLC method met the requirements for biological sample determination. The cytotoxicity of vancomycin in HK-2 cells was concentration-dependent within 1–20 mg/mL; omeprazole could reduce the intracellular accumulation of vancomycin. Western blot assay confirmed that omeprazole increased intracellular vancomycin efflux by upregulating P-gp expression and inhibited its intracellular transport by downregulating OCT-2 expression. Vancomycin increased renal function indicators and pathological injury score in rats, while there was a significant decrease in the drug combination group, together with alleviated renal tissue damage.

**Conclusion:** Intracellular accumulation of vancomycin can cause damage to HK-2 cells and induce vancomycin-related nephrotoxicity in rats. Omeprazole can reduce vancomycin cytotoxicity by upregulating P-gp expression and inhibiting OCT-2 expression.

## 1. Introduction

Vancomycin is the first-line treatment of methicillin-resistant *Staphylococcus aureus* (MRSA) infections. It has no cross-resistance with other antibiotics, and the induced resistance of MRSA to vancomycin is very low [1]. Vancomycin can be used for sepsis, infectious endocarditis, osteomyelitis, pneumonia, lung abscess, peritonitis, and meningitis with definite clinical efficacy [2, 3]. However, there are significant individual differences in vancomycin pharmacokinetics, especially in children, older adults, and critically ill patients [4–7]. Excessive vancomycin blood concentration can cause nephrotoxicity [8].

Vancomycin is excreted primarily as the prototype drug through glomerular filtration, and its excretion-related transporters include organic cation transporter (OCT)-2 and P-glycoprotein (P-gp). OCT-2 is the main OCTs in the kidneys, which participate in the renal excretion of organic cations, cationic drugs, and toxins in the body, as well as the reabsorption of some endogenous substances and drugs after glomerular filtration [9, 10]. OCT-2 is closely related to the pharmacokinetic process and nephrotoxicity of drugs. Upregulation of OCT-2 expression can increase drug reabsorption in the kidneys, leading to drug accumulation in renal tubular epithelial cells and causing nephrotoxicity. P-gp is a drug efflux pump primarily expressed on the apical membrane of renal proximal tubular (HK-2) cells, which can rely on energy to pump drugs out of the cell. Downregulation of P-gp expression can lead to reduced drug excretion, thereby exacerbating renal injury [11]. Reducing drug accumulation in HK-2 cells can effectively alleviate vancomycin-induced renal injury [12, 13].

Studies have shown that cilastatin can reduce vancomycin accumulation and nephrotoxicity in renal tubular epithelial cells by inhibiting the “endocytosis protein” [14, 15]. Unfortunately, cilastatin significantly reduces vancomycin concentration in plasma and potentially diminishing the antibacterial effect of vancomycin [11]. Therefore, we tried to find a better drug to alleviate vancomycin-induced nephrotoxicity without lowering the plasma vancomycin concentration. Omeprazole was reported to reduce cisplatin uptake by HK-2 cells by inhibiting OCT-2 activity and increasing cisplatin efflux by regulating P-gp [16]. The process reduced the accumulation of cisplatin in HK-2 cells, minimizing drug-induced nephrotoxicity, and did not lead to a reduction of plasma cisplatin concentration [17]. Another study suggested that omeprazole could attenuate cisplatin-induced kidney injury through suppression of the TLR4/NF-kappaB/NLRP3 signaling pathway [18]. However, clinical studies have raised that long-term use of omeprazole was associated with the morbidity of chronic kidney disease [19, 20], and animal study has concluded that the long-term use of omeprazole resulted in structural damage of rat renal tissue in a time-dependent manner [21].

Based on previous studies, we selected omeprazole as the intervention drug for short-term use. We aimed to clarify the effects of omeprazole on vancomycin-induced cytotoxicity by studying the activity and morphology of HK-2 cells, analyzing the concentration of vancomycin in HK-2 cells, and performing pathological examination of kidney tissue of rats. Western blot was used to investigate changes in the protein expression of P-gp and OCT-2 transporters in HK-2 cells to explore the protective mechanism of omeprazole against vancomycin-induced cell damage.

## 2. Material and Methods

### 2.1. Instruments

The following instruments were used: Waters E2695 HPLC system (Empower Application operating platform, Waters, United States), Diamonsil C18 column (150 mm × 4.6 mm, 5 *μ*m), ultrasonic cell disruptor (JY92, Ningbo Xinzhi Biotechnology Co. Ltd., China), microplate reader (Multiskan FC model, Thermo Fisher Scientific, United States), inverted fluorescence microscope (Axio Vert.A1, Carl Zeiss Microscopy GmbH, Germany), gel imaging system (Universal Hood III, Bio-Rad, United States), and horizontal electrophoresis apparatus (DYCP-31, Bio-Rad, United States).

### 2.2. Drugs and Reagents

Vancomycin reference substance (content 98.90%, 130360) was obtained from the China National Institute for Food and Drug Control). Vancomycin hydrochloride for injection (500 mg, HJ20140174) was procured from Vianex S.A., Greece. Omeprazole sodium for injection (40mg, H20033394) was purchased from AstraZeneca Pharmaceutical Co. Ltd. E-MEM medium (500 mL, 01-026-1) was supplied by BI Biopharmaceutical Co. Ltd., United States. A CCK-8 kit (500 T, ZP328-2) was acquired from Beijing Zhuangmeng International Bio-Gene Technology Co. Ltd. A BCA protein concentration assay kit (50 T, PC0020) was obtained from Solarbio. Antibodies included P-gp Rabbit mAb (20 *μ*L, R25231, Chengdu Zhengneng Biotechnology Co. Ltd., China) and OCT-2 Rabbit pAb antibody (50 *μ*L, 121071, Chengdu Zhengneng Biotechnology Co. Ltd., China). Detection kits were as follows: blood urea nitrogen (BUN) detection kit (C013-2-1, Nanjing Jiancheng Biotechnology Research Institute), total protein quantitative assay kit (A045-2-2, Nanjing Jiancheng Biotechnology Research Institute), blood creatinine (CRE) detection kit (C011-2-1, Nanjing Jiancheng Biotechnology Research Institute), *N*-acetyl-*β*-d-glucosaminidase (NAG) detection kit (A031-1-1, Nanjing Jiancheng Biotechnology Research Institute), and kidney injury molecule-1 (KIM-1) ELISA Kit (E-EL-R3019, Wuhan Elirit Biotechnology Co. Ltd.).

### 2.3. Cell Lines and Experimental Animal

HK-2 cells were acquired from Wuhan Procell Life Science & Technology Co. Ltd., China). Male SD rats (7–8 weeks, weighing 200–220 g) were obtained from Beijing Huafukang Experimental Animal Co. Ltd. This study has been approved by the Ethics Committee of the Second Hospital of Hebei Medical University (No 2024-AE004).

### 2.4. Effect of Omeprazole on Vancomycin-Induced HK-2 Cytotoxicity

#### 2.4.1. Inhibitory Effect of Vancomycin on HK-2 Cells

HK-2 cells were inoculated in a 96-well plate (5 × 10^3^ cells per well). E-MEM medium containing 10% fetal bovine serum and 1% penicillin–streptomycin was added to wells. Cells were incubated at 37°C in a 5% CO_2_ incubator for 24 h to allow the cells to adhere to the wall and were cultured for 18 h. Complete culture medium (100 *μ*L) containing different concentrations of vancomycin (1, 2, 4, 8, 12, 15, 18, and 20 mg/mL) was added sequentially and incubated for 24 h. The CCK-8 reagent (10 *μ*L) was then added to react for 4 h. OD values were measured by a microplate reader. The cell survival and inhibitory rates of different vancomycin concentrations on HK-2 cells were calculated according to the following equation:

 $$\begin{array}{c} \text{Inhibitory rates}=\frac{\text{Ac}-\text{As}}{\text{Ac}-\text{Ab}}\times 100\% \end{array}$$

where *A*_c_ is the absorbance of the cell survival well, *A*_s_ is the absorbance of the intervention well, and *A*_b_ is the absorbance of the blank control well.

#### 2.4.2. Protective Effect of Omeprazole on Vancomycin-Induced HK-2 Cytotoxicity

The effect of the drug on HK-2 cells was determined according to Section 2.4.1: (1) the control group, in which blank culture medium was added to each well; (2) the vancomycin group, in which complete culture medium containing 4 mg/mL vancomycin was added; and (3) the drug combination group, which contained 4 mg/mL vancomycin and omeprazole at low (5 *μ*g/mL), medium (50 *μ*g/mL), and high (100 *μ*g/mL) concentrations, respectively. After 24 h of intervention, the morphology of cells was observed by an inverted fluorescence microscope. CCK-8 reagent (10 *μ*L) was added to each well. After 4 h of reaction, the OD values were measured using a microplate reader. The drug's effect on the activity of HK-2 cells was assessed by cell numbers and survival rates.

### 2.5. Determination of Vancomycin Concentration in HK-2 Cells

#### 2.5.1. Chromatographic Conditions

Chromatographic separation was performed on a Diamonsil C18 column (150 mm × 4.6 mm, 5 *μ*m). The mobile phase consisted of methanol and 0.05 mol/L potassium dihydrogen phosphate buffer (pH = 3) (17: 83, V/V). The flow rate was maintained at 1.0 m/min, and the column temperature was set to 30°C. Detection was performed at a wavelength of 236 nm, with an injection volume of 20 *μ*L.

#### 2.5.2. Sample Handling

The culture medium of HK-2 cells in the logarithmic growth period was taken and centrifuged (13,000 r/min) at 4°C for 20 min. The upper extracellular fluid was removed and stored at −40°C. The surfaces of HK-2 cells were washed with PBS, and the cell sediments were collected after trypsin digestion and centrifugation. The sediments were cleaned and resuspended with PBS (3 × 10^5^/mL) and repeatedly frozen and thawed five times at −80°C and 37°C, respectively. Cells were broken by ultrasound and centrifuged at 4°C for 20 min at 13,000 r/min. The upper intracellular fluid was removed and stored at −40°C.

For intracellular and extracellular fluids, 200 *μ*L of each fluid was taken and added to 400 *μ*L methanol. The solution was mixed by vortex and centrifuged at 13,000 r/min, 4°C for 20 min. The supernatant was filtered through a 0.22-*μ*m membrane and then injected for analysis.

#### 2.5.3. Specificity

Blank intracellular and extracellular fluids and vancomycin-containing intracellular and extracellular fluids were processed according to “sample treatment.” Samples were injected, and chromatograms were recorded to investigate the technique's specificity.

#### 2.5.4. Standard Curve

Extracellular and intracellular fluid samples containing vancomycin in a series of concentrations (0.001, 0.01, 0.1, 0.5, 1, 2, 4, and 8 mg/mL) were separately prepared. Samples were processed according to “sample handling.” Using vancomycin concentration (*X*) as abscissa and peak area (*Y*) as the ordinate, the weighted least square method (1/*X*^2^) was used for the linear regression evaluation. The standard curves of vancomycin intracellular solution and extracellular solution were drawn, respectively.

#### 2.5.5. Recovery and Precision

Five intracellular solution samples and five extracellular solutions with low (0.01 mg/mL), medium (1 mg/mL), and high (6.4 mg/mL) vancomycin concentrations were prepared, processed, and measured according to “sample handling.” Vancomycin concentrations were calculated on the standard curve. The absolute recovery rate was calculated as the ratio of the measured concentration to the concentration measured by direct injection of the same amount of vancomycin reference solution. The relative recovery was calculated by the ratio of the measured concentration to the theoretical concentration.

Similarly, five intracellular solution samples and five extracellular solutions of the low, medium, and high vancomycin concentrations were prepared. The samples were processed and determined within 1 day according to “sample handling” to calculate the intraday precision. The samples were processed and measured within 3 days to calculate the interday precision.

#### 2.5.6. Stability

Five intracellular solution samples and five extracellular solutions of the low, medium, and high vancomycin concentrations were prepared, stored at −40°C for 3 days, or repeatedly frozen and thawed three times between −40°C and room temperature. Samples were processed and measured based on “sample handling.” The low-temperature storage and repeated freeze-thaw stability of the samples were investigated.

### 2.6. Effect of Omeprazole on Vancomycin Concentration in HK-2 Cells

HK-2 cells were incubated in the cell incubator up to the logarithmic growth stage. Drug intervention was carried out after culture starvation: (1) vancomycin group, in which complete culture medium containing 4 mg/mL vancomycin was added to each double-well, and (2) drug combination group, in which omeprazole (5, 50, and 100 *μ*g/mL) and vancomycin complete culture medium (4 mg/mL) were added, respectively. After 24 h of intervention, each group was treated and measured according to “sample handling.” The effects of omeprazole concentrations on the vancomycin concentrations in HK-2 intracellular and extracellular fluids were investigated.

### 2.7. Effect of Omeprazole on the Expression of P-gp and OCT-2 Transporters in HK-2 Cells

After culture starvation, HK-2 cells in the logarithmic growth phase were divided into two groups: (1) vancomycin group, which consisted of a complete culture medium containing different vancomycin concentrations (0, 4, 6, and 8 mg/mL), and (2) drug combination group, which consisted of omeprazole (5, 50, and 100 *μ*g/mL) with vancomycin complete culture medium (4 mg/mL).

After the intervention in each group, the cell surface was cleaned with PBS. The target protein was extracted, lysed with a RIPA buffer containing 1% phenylmethane sulfonyl fluoride (PMSF) in an ice bath, and centrifuged at 4°C for 20 min. Using the BCA method, the supernatant was removed to determine the total protein concentration.

The protein lysate was mixed with 5× loading buffer in a 4:1 ratio and heated in a constant temperature metal bath at 100°C for 10 min. The mixture was cooled and separated by SDS polyacrylamide gel electrophoresis and transferred to the PVDF membrane. The membrane was sealed with 5% skim milk. The P-gp and OCT-2 antibodies were added and incubated overnight at 4°C. The corresponding horseradish peroxidase binding secondary antibody was added and incubated at room temperature for 1 h. The bands were cleaned using TBST and exposed to ECL reagent. Image Lab and ImageJ software were used to quantitatively detect the protein band's relative strength.

### 2.8. Effect of Omeprazole on Vancomycin-Related Renal Injury in Rats

#### 2.8.1. Grouping and Drug Administration

Thirty rats were randomly divided into three groups: control group, vancomycin group, and drug combination group, with 10 rats in each group. Each rat in the vancomycin group was given 400 mg/kg vancomycin intraperitoneally, the drug combination group was given 400 mg/kg vancomycin intraperitoneally and 4 mg/kg omeprazole intravenously, and the control group was given equal volume of 0.9% sterile sodium chloride, qd, for 7 consecutive days.

#### 2.8.2. Determination of Renal Injury Indicators and Renal Tissue Pathology

Before daily administration, blood was collected from the internal horn vein of each rat, and the contents of BUN and blood CRE were determined after centrifugation. On the second day after continuous administration for 7 days, the rats were euthanized, and their kidneys were removed. One part of kidney tissue was taken and homogenized to determine the expression of NAG and KIM-1. Another part of kidney tissue was fixed with 4% paraformaldehyde, trimmed, dehydrated, embedded, sliced, stained with HE, and sealed for pathological examination and renal injury score according to the International Harmonization of Nomenclature and Diagnostic Criteria (INHAND) [22].

### 2.9. Statistical Analysis

All data were expressed as mean ± standard deviation (SD). The correlation between the intracellular vancomycin concentration and HK-2 cell injury was analyzed by one-way ANOVA. Firstly, normality tests were conducted on the data, and paired sample *t*-tests were used for data that followed a normal distribution to analyze the effects of omeprazole on vancomycin concentration and the expression of P-gp and OCT-2 transporters in HK-2 cells. *p* < 0.05 is statistically significant. IBM SPSS Statistics 23 software was used for statistical analysis.

## 3. Results

### 3.1. Effect of Omeprazole on Vancomycin-Induced Cytotoxicity of HK-2 Cells

#### 3.1.1. Cell Survival

**Figure 1:**
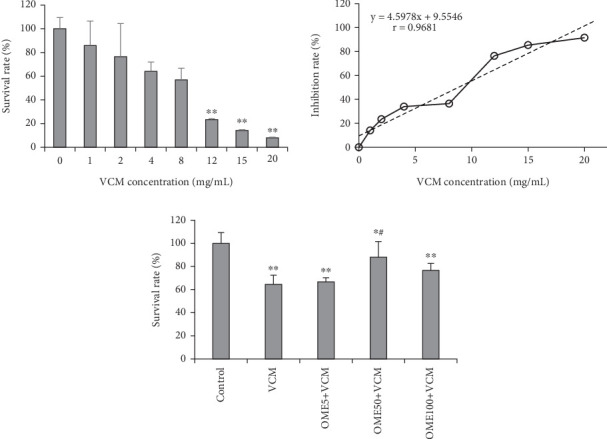
Effects of vancomycin (VCM) and omeprazole (OME) on the survival rate of HK-2 cells. OME5: 5 *μ*g/mL OME; OME50: 50 *μ*g/mL OME; OME100: 100 *μ*g OME; ⁣^∗^*p* < 0.05, ⁣^∗∗^*p* < 0.01, compared with the control group; ⁣^#^*p* < 0.05, compared with the VCM group.

Vancomycin had an apparent toxic effect on HK-2 cells in the range of 1–20 mg/mL. The degree of cell damage was proportional to vancomycin concentration within the intracellular fluid. When vancomycin concentration exceeded 12 mg/mL, it significantly inhibited the activity of HK-2 cells (*p* < 0.05, IC50 = 8.8 mg/mL). Omeprazole could effectively reduce the vancomycin cytotoxicity in HK-2 cells. Compared with the vancomycin group, the survival rate of HK-2 cells increased significantly after omeprazole (50 *μ*g/mL) was added (*p* < 0.05). The results are shown in Figure 1.

#### 3.1.2. Cell Morphology

**Figure 2:**
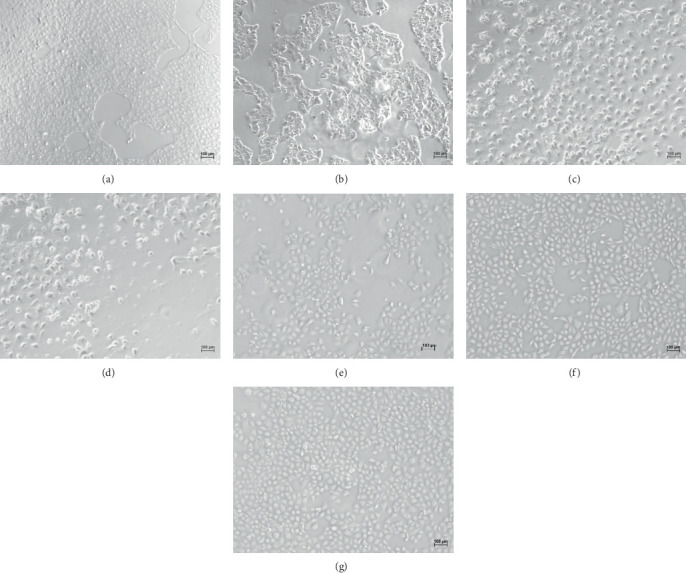
Effects of vancomycin (VCM) and omeprazole (OME) on the morphology of HK-2 cells. (a) Control. (b) 2 mg/mL VCM. (c) 4 mg/mL VCM. (d) 6 mg/mL VCM. (e) 5 *μ*g/mL OME + 4 mg/mL VCM. (f) 50 *μ*g/mL OME + 4 mg/mL VCM. (g) 100 *μ*g/mL OME + 4 mg/mL VCM.

The results of the morphological observation of HK-2 cells under the microscope are shown in Figure 2. Normal HK-2 cells were closely connected to the wall, and the shape was plump and spherical, with scattered floating cell debris (Figure 2a). The cell morphology changed with vancomycin concentration. When vancomycin was 2 mg/mL, HK-2 cells were scattered and connected under the microscope. The cell surface was shrunken and damaged with floating cell debris (Figure 2b). The cell junction area was significantly reduced when vancomycin was 4 mg/mL. The shape was contracted and ruptured with a large amount of floating cell debris (Figure 2c). The number of adherent cells was significantly reduced when the vancomycin concentration increased to 6 mg/mL. The cell surface was reduced, and the morphology was broken with increased floating cell debris (Figure 2d). Compared to the vancomycin group, the cell morphology of the drug combination group (5 *μ*g/mL omeprazole) was improved. There was no apparent shrinkage or damage (Figure 2e). The cell morphology was close to that of the control group when the omeprazole concentration increased to 50 and 100 *μ*g/mL. The number of tight junctions increased, and the shape was plump and round (Figure 2f,g).

### 3.2. Effect of Omeprazole on Vancomycin Concentration in HK-2 Cells

#### 3.2.1. Specificity

The HPLC diagrams of the intracellular and extracellular fluids of HK-2 showed that the retention time of vancomycin was 17.1 min. The separation was completed with good efficacy. Endogenous substances and culture medium did not interfere with the determination of vancomycin in the sample. The HPLC chromatograms of HK-2 intracellular fluid and extracellular fluid are shown in Figure S1 in supplementary material.

#### 3.2.2. Standard Curve

Under the selected chromatographic conditions, the vancomycin standard curve regression equation is *Y* = 6.20*X* − 0.00168 (*r* = 0.9974) in the intracellular fluid and *Y* = 5.12*X* + 0.00343 (*r* = 0.9990) in the extracellular fluid. The linear relationship was good in the 0.001–8 mg/mL range.

#### 3.2.3. Recovery and Precision

The absolute recovery rates of vancomycin in intracellular and extracellular fluids were 79.81%–93.97% and 92.75%–97.78%, respectively. The relative recovery rates were 84.98%–97.30% and 85.33%–99.84%, respectively. The relative standard deviation (RSD) values of intraday and interday precision were less than 10%. The results are shown in Table 1.

#### 3.2.4. Stability

Three vancomycin concentrations in intracellular and extracellular fluids were stable when stored at −40°C for 3 days or repeatedly frozen-thawed three times. RSD values were less than 10% (Table 2).

#### 3.2.5. Effect of Omeprazole on Vancomycin Concentration in HK-2 Cells

Compared to the vancomycin group, the accumulation of vancomycin in HK-2 cells decreased after omeprazole was added. The vancomycin content in the extracellular fluid was proportional to the omeprazole concentration. On the contrary, the lower the vancomycin concentration in the intracellular fluid sample, the higher the vancomycin concentration in the corresponding extracellular fluid sample. The results are shown in Table 3.

### 3.3. Effect of Omeprazole on the Expression of P-gp and OCT-2 in HK-2 Cells

**Figure 3:**
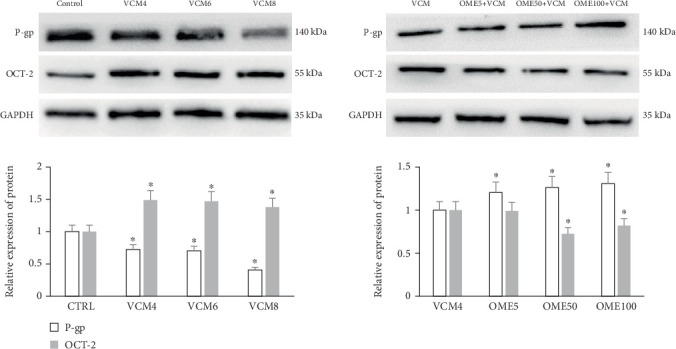
Effect of vancomycin (VCM) on the protein expression of P-gp and OCT-2 in HK-2 cells. VCM 4: 4 mg/mL VCM; VCM 6: 6 mg/mL VCM; VCM 8: 8 mg/mL VCM; OME5: 5 *μ*g/mL OME; OME50: 50 *μ*g/mL OME; OME100: 100 *μ*g/mL OME; ⁣^∗^*p* < 0.05, compared with the control group.

The protein expression results of the P-gp and OCT-2 transporters in HK-2 cells are shown in Figure 3. The expression of the target protein in HK-2 cells in the vancomycin group was significantly different from that of the control group. The expression of P-gp decreased with increasing vancomycin concentration (*p* < 0.05). However, the protein expression of OCT-2 was significantly upregulated (*p* < 0.05).

Compared to the vancomycin group, the expression of P-gp in HK-2 cells was significantly increased after combined with omeprazole (*p* < 0.05). Compared to vancomycin group, there were no significant differences in OCT-2 protein expression in the combined group (omeprazole 5 *μ*g/mL, *p* > 0.05). The expression of OCT-2 was significantly downregulated (*p* < 0.05) in the combined groups of omeprazole (50 and 100 *μ*g/mL).

### 3.4. Effect of Omeprazole on Vancomycin-Related Renal Injury in Rats

**Figure 4:**
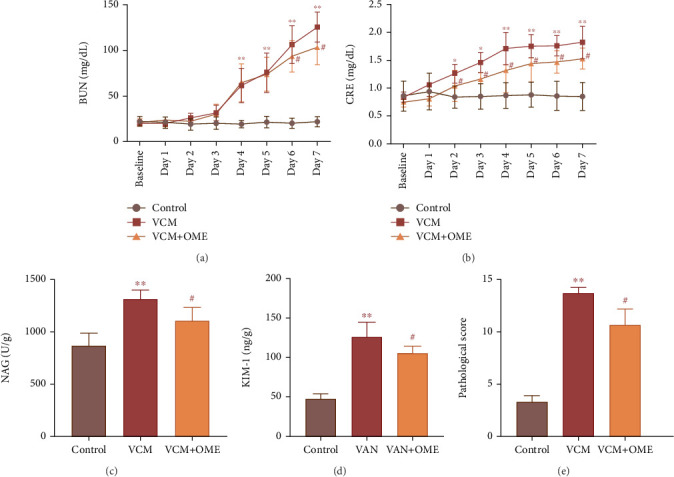
Comparison of renal function indicators and renal injury markers in rats. (a) BUN. (b) CRE. (c) NAG. (d) KIM-1. (e) Renal injury score. VAN: 400 mg/kg VAN; VAN + OME: 400 mg/kg VAN + 4 mg/kg OME; ⁣^∗^*p* < 0.05, ⁣^∗∗^*p* < 0.01 compared to the control group; ⁣^#^*p* < 0.05 compared to the VAN group.

The comparison of renal function indicators and renal injury markers in different groups is shown in Figure 4. Compared with the control group, the levels of NAG, KIM-1, and pathological injury score of the vancomycin group were significantly increased (*p* < 0.01), and the BUN values in d4–d7 and CRE values in d4–d7 were significantly higher than the control group (*p* < 0.01). Compared with the vancomycin group, the drug combination group showed a significant decrease in NAG, KIM-1 levels, and pathological injury score (*p* < 0.05), with BUN values in d6–d7 and CRE values in d2–d7 significantly lower than the vancomycin group (*p* < 0.05).

**Figure 5:**
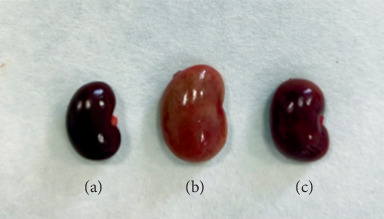
The images of the kidneys of rats. (a) Control group. (b) VCM group. (c) Drug combination group.

**Figure 6:**
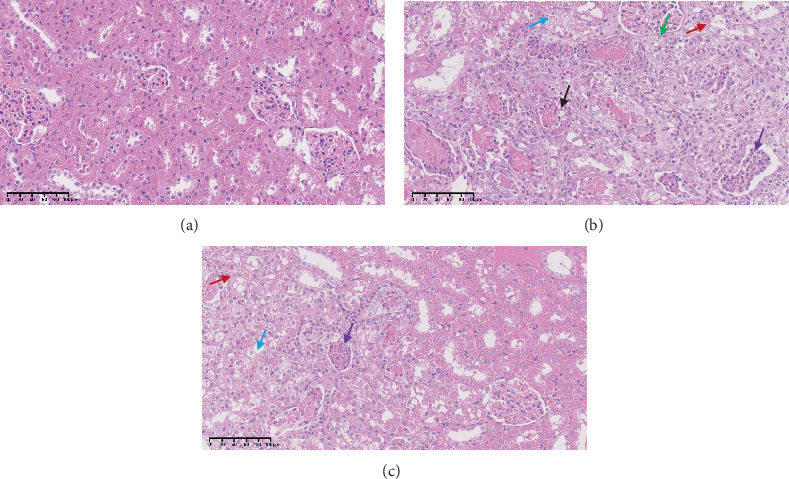
Pathological sections of kidney tissue of rats. (a) Control group. (b) VCM group. (c) Drug combination group. The red arrow represents vacuolar degeneration, the blue arrow represents watery degeneration, the black arrow represents tubular necrosis, the green arrow represents tubular atrophy, and the purple arrow represents inflammatory cell infiltration.

The images of the kidneys of rats in three groups are shown in Figure 5. Compared with the control group, the kidneys of rats in the vancomycin group showed significant swelling, increased volume, and lighter color. Compared with the vancomycin group, the drug combination group showed a decrease in kidney size and reduced edema, and the kidney volume was similar to that of the control group. The pathological sections of kidney tissue of rats are shown in Figure 6. The vancomycin group showed a large amount of watery degeneration of renal tubular epithelial cells in the cortex. Multiple renal tubules were necrotic, with necrotic cell fragments and eosinophils in the lumen. While in the drug combination group, only some renal tubular epithelial cells showed watery degeneration. Individual renal tubules were necrosis. Compared with the vancomycin group, the drug combination group showed alleviated renal tissue damage.

## 4. Discussion

Drug-resistant bacterial infection is a critical challenge for clinical anti-infection treatment [23]. Vancomycin is widely used in practice as the first-line treatment for MRSA infections. However, vancomycin adverse reactions, especially nephrotoxicity, affect the safety of clinical drug treatment. Therapeutic drug monitoring (TDM) is recommended in patients concurrently taking other nephrotoxic drugs and newborns and infants [24–27]. Individualized drug use through TDM is essential to improve curative effects and reduce adverse reactions. Another crucial clinical pharmacy research is to study the mechanisms of drug-induced nephrotoxicity and take the corresponding measures.

The causes of vancomycin nephrotoxicity are complex and diverse, among which the accumulation of drugs in renal tubular epithelial cells and the induction of oxidative stress are the main reasons [28]. Increased serum and intracellular drug concentrations are related to vancomycin nephrotoxicity [29]; the risk of vancomycin-related acute renal injury increases when the vancomycin peak concentration is > 20 *μ*g/mL [24]. Based on the clinical practice, the target trough concentration range for vancomycin was 10–20 *μ*g/mL [24], and the peak concentration was up to 50–80 *μ*g/mL [30–32], considering that the concentration of vancomycin in renal tubules is about 40–50 times that of plasma [33]. We selected the range of 1–20 mg/mL to investigate the cytotoxicity of vancomycin on HK-2 cells in this study.

Many drugs can alleviate drug-induced kidney damage by reducing the concentration of drugs in the renal tissue. Thymoquinone and selenide can significantly inhibit oxidative damage, apoptosis, and the inflammatory response by promoting methylation and excretion of sodium arsenite in renal tissues [34]. Cyclo-trans-4-l-hydroxypropyl-l-serine can reverse the effect of vancomycin on OCT-2 and P-gp, improve the pathological condition and morphology of the rat kidneys, and alleviate renal injury caused by vancomycin [35]. l-Tetrahydropalmatine reduces cisplatin-induced nephrotoxicity by selectively inhibiting OCT-2 without affecting its antitumor efficacy [36]. The proton pump inhibitors omeprazole and rabeprazole can minimize the accumulation of cisplatin in HK-2 cells by regulating the expression of P-gp and OCT-2, thus alleviating the nephrotoxicity of cisplatin [18, 37]. Based on the regulatory effects of vancomycin on P-gp and OCT-2, omeprazole chose was selected as the intervention drug in this study. Considering the association between long-term use of omeprazole and kidney injury in animal experiments and clinical studies, we chose short-term administration of omeprazole. In the HK-2 cell study, the intervention time of omeprazole was 24 h, and in the in vivo study of rats, the administration time was 7 days, and no omeprazole-related renal damage was found.

Through cell proliferation inhibition tests and morphological investigation, the survival rate of HK-2 cells gradually decreased with the increase of vancomycin concentration, indicating that vancomycin HK-2 cytotoxicity was dose-dependent. The combined use of omeprazole reversed the vancomycin-induced HK-2 cytotoxicity. Animal experiment showed that omeprazole can reverse the increase of vancomycin-related renal function indicators, BUN, CRE, NAG, and KIM-1, and alleviate the edema of renal tubular epithelial cells and tubular necrosis. The experiments confirmed that omeprazole had a protective effect on vancomycin-associated renal injury.

Drug combinations for reducing vancomycin nephrotoxicity were investigated through changes in biochemical indicators related to renal function in experimental animals or humans in previous studies [38–40]. There has been no research report on the change in intracellular drug concentration before and after the combination of drugs. In this study, while investigating the changes in HK-2 cell activity, the changes in vancomycin concentration in HK-2 cells were compared. The results showed a decrease in the concentration and accumulation of vancomycin in HK-2 cells after combining with omeprazole. The findings were further verified through animal experiments and confirmed the protective effect of omeprazole on the damage of HK-2 cells induced by vancomycin.

## 5. Conclusion

The toxic effect of vancomycin on HK-2 cells increased in a concentration-dependent manner. Omeprazole could upregulate the expression of the efflux transporter P-gp and inhibit the intracellular transport of drugs by OCT-2, reduce vancomycin concentration in HK-2 cells, and relieve vancomycin cytotoxicity. Animal experiments demonstrated that omeprazole could reverse the high expression of renal injury markers related to vancomycin, alleviate edema of renal tubular epithelial cells and tubular necrosis, and perform a protective effect on vancomycin-induced renal injury.

## Figures and Tables

**Table 1:** Recovery and precision of vancomycin in intracellular and extracellular fluids (*n* = 5).

**Conditions**	**Concentration (mg/mL)**	**Absolute recovery**		**Relative recovery**		**Intraday precision**		**Interday precision**	
		**M** **e** **a** **n** ± **SD (%)**	**RSD (%)**	**M** **e** **a** **n** ± **SD (%)**	**RSD (%)**	**Measured (mg/mL)**	**RSD (%)**	**Measured (mg/mL)**	**RSD (%)**
Intracellular fluid	0.01	79.81 ± 0.87	1.09	85.47 ± 1.70	1.99	0.0114 ± 0.00048	5.18	0.0114 ± 0.0009	8.65
	1	93.97 ± 2.48	2.64	97.30 ± 1.41	1.45	1.09 ± 0.04	3.61	1.09 ± 0.16	14.59
	6.4	88.23 ± 3.99	4.52	84.98 ± 3.93	4.62	6.67 ± 0.40	6.01	6.95 ± 0.35	5.02

Extracellular fluid	0.01	92.75 ± 4.11	4.43	85.33 ± 4.14	4.86	0.0105 ± 0.00088	8.37	0.0103 ± 0.00097	9.38
	1	97.09 ± 4.13	4.25	99.84 ± 3.38	3.38	1.10 ± 0.05	4.49	1.11 ± 0.15	13.36
	6.4	97.78 ± 0.64	0.65	94.50 ± 0.60	0.64	6.47 ± 0.21	3.24	6.52 ± 0.27	4.11

Abbreviations: RSD, relative standard deviation; SD, standard deviation.

**Table 2:** Stability of vancomycin in intracellular and extracellular fluids (*n* = 5).

**Treatment**	**Concentration (mg/mL)**	**Intracellular fluid**		**Extracellular fluid**	
		**Founded (mg/mL)**	**RSD (%)**	**Founded (mg/mL)**	**RSD (%)**
Storage at −40°C for 3 days	0.01	0.0113 ± 0.0003	2.55	0.0103 ± 0.0006	6.13
	1	1.12 ± 0.03	2.57	1.04 ± 0.02	1.79
	6.4	6.83 ± 0.10	1.50	6.64 ± 0.34	5.18

Three freeze-thaw cycles	0.01	0.0109 ± 0.0009	8.13	0.0105 ± 0.0010	9.36
	1	0.90 ± 0.02	2.20	0.94 ± 0.03	2.83
	6.4	6.35 ± 0.24	3.75	6.88 ± 0.07	1.02

Immediately determine	0.01	0.0105 ± 0.0004	3.75	0.0105 ± 0.0007	6.42
	1	1.05 ± 0.01	1.04	1.12 ± 0.03	2.80
	6.4	6.51 ± 0.54	8.23	6.37 ± 0.05	0.78

Abbreviation: RSD, relative standard deviation.

**Table 3:** Effects of omeprazole on the vancomycin concentration in HK-2 cells.

**Omeprazole concentration (*μ*g/mL)**	**Vancomycin concentration (mg/mL)**	
	**Intracellular fluid**	**Extracellular fluid**
0	2.41	2.55
5	1.05⁣^∗^	2.80⁣^∗^
50	0.41⁣^∗∗^	2.82⁣^∗^
100	0.39⁣^∗∗^	2.83⁣^∗^

⁣^∗^*p* < 0.05, ⁣^∗∗^*p* < 0.01, compared with the VCM group.

## Data Availability

The data that support the findings of this study are available from the corresponding authors upon reasonable request.

## Supporting Information

Supporting Information 1

Supporting Information 2 Figure S1: HPLC chromatogram of HK-2 intracellular and extracellular fluid. (a) Intracellular fluid. (b) Extracellular fluid. (c) Vancomycin. (d) Intracellular fluid with vancomycin. (e) Extracellular fluid with vancomycin. 1: Vancomycin.
