# Supplementary material for: The Protective Effect of Omeprazole on Vancomycin Cytotoxicity in HK-2 Cells and Renal Injury in Rats
Source: Biomed Res Int. 2025 Jul 31;2025:3520935. doi: 10.1155/bmri/3520935 (PMC12401607; doi:10.1155/bmri/3520935)
Supplement: Supporting Information 2 — Figure S1: HPLC chromatogram of HK-2 intracellular and extracellular fluid. (a) Intracellular fluid. (b) Extracellular fluid. (c) Vancomycin. (d) Intracellular fluid with vancomycin. (e) Extracellular fluid with vancomycin. 1: Vancomycin. [file 3520935.f2.docx]

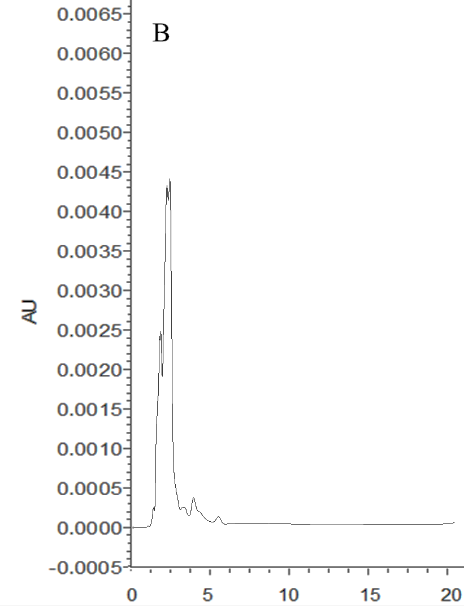

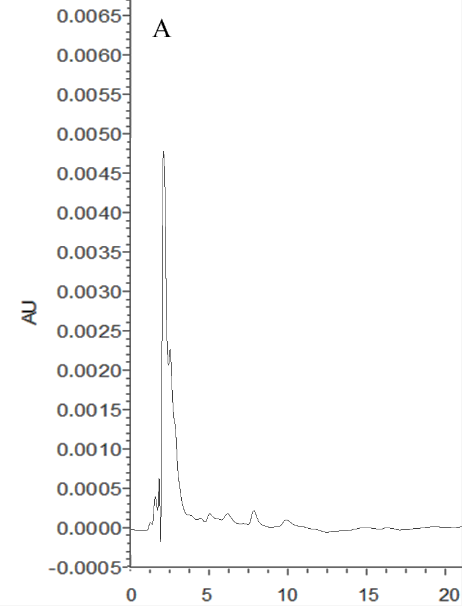

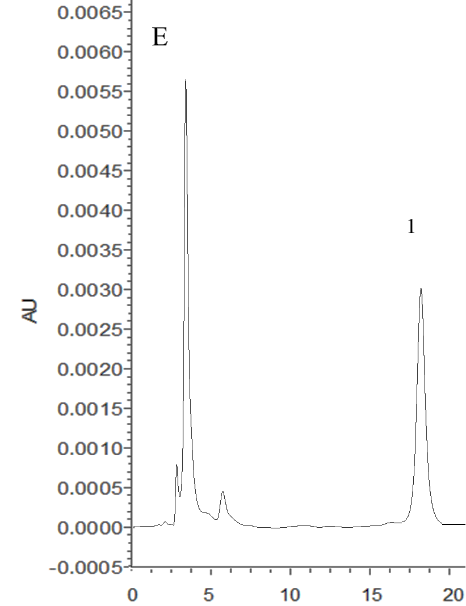

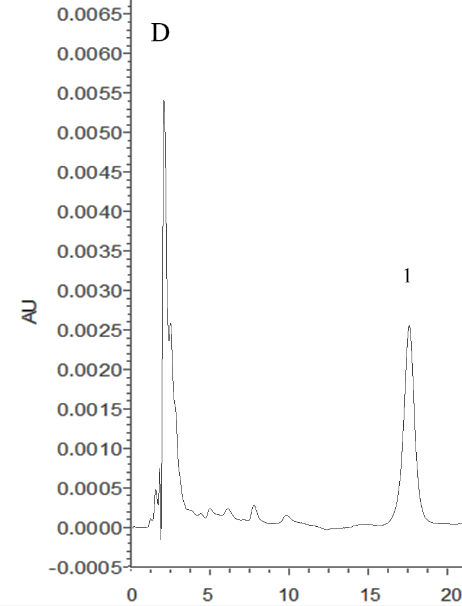

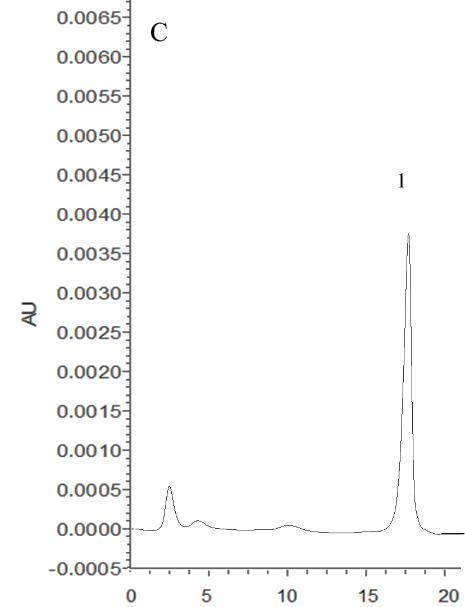


Fig.7 HPLC chromatograms of HK-2 intracellular and extracellular fluids

A: Intracellular fluid; B: Extracellular fluid; C: Vancomycin; D: Intracellular fluid with vancomycin; E: Extracellular fluid with vancomycin; 1: Vancomycin
